# Clinical Efficacy of Polycaprolactone β-Calcium Triphosphate Composite for Osteoconduction in Rabbit Bone Defect Model

**DOI:** 10.3390/polym13152552

**Published:** 2021-07-31

**Authors:** Chiu-Ming Chen, Shen-Mao Chen, Shiou-Fu Lin, Huang-Chien Liang, Chia-Chun Wu

**Affiliations:** 1 Department of Orthopaedics, Tri-Service General Hospital, National Defense Medical Center, Taipei City 11490, Taiwan; ccm20349@gmail.com (C.-M.C.); smchen1113@gmail.com (S.-M.C.); 2 Department of Pathology, Shuang-Ho Hospital, Taipei Medical University, Taipei City 23561, Taiwan; 18011@s.tmu.edu.tw; 3 Department of Materials Engineering, Ming Chi University of Technology, New Taipei City 24301, Taiwan; 930747@gmail.com

**Keywords:** β-tricalcium phosphate, biocomposites, osteoconduction, polycaprolactone

## Abstract

The combination of β-tricalcium phosphate (β-TCP) with polycaprolactone (PCL) has been considered a promising strategy for designing scaffolds for bone grafting. This study incorporated PCL with commercially available β-TCP (Osteocera^TM^) to fabricate an injectable bone substitute and evaluate the effect of PCL on compressive strength and setting time of the hydraulic cement. The mechanical testing was compliant with the ASTM D695 and ASTM C191-13 standards. Results showed that PCL-TCP composite presented a well-defined architecture with uniform pore distribution and a significant increase in compressive strength compared with β-TCP alone. Eighteen rabbits, each with two surgically created bone defects, were treated using the PCL-TCP composites. The composite materials were resorbed and replaced by newly formed bone tissue. Both PCL-TCP and β-TCP demonstrated equivalent clinical effects on osteoconduction property in terms of the percentage of newly formed bone area measured by histomorphometric analysis. PCL-TCP was proven to be as effective as the commercially available β-TCP scaffold (Osteocera^TM^).

## 1. Introduction

Repair of large bone defects remains an unmet clinical need in modern orthopedics. These defects have resulted in poor quality of life for aging populations and have become a growing socioeconomic concern around the world [1]. Bone regeneration occurs with a balance of signaling events in osteoblasts, bone-forming cells and osteoclasts to restore bone structure and function [2]. Natural bone is made of inorganic and organic components such as extracellular matrix (ECM) and calcium phosphate ceramics [3]. To address the continuous resorption of bone because of age or disease, use of biomaterials that are compatible with bone and are inert to the immune system has been developed.

Biomaterials such as polymers, metals and ceramics used in clinical applications of orthopedics and dentistry procedures have been known to contain several disadvantages. Synthetic and biodegradable polymers with tunable properties used as bone scaffolds have been reported to raise the risk of immunogenicity and toxicity [4,5]. Metals such as titanium (Ti), magnesium and stainless steel possess great mechanical strength and excellent fatigue resistance. However, they can have poor integration properties with surrounding tissues and may release metal ions [6,7]. Currently, use of calcium phosphate-based biomaterials is considered as the gold standard [8].

Depending on osteoinduction, an ability to induce osteoblastic differentiation and osteoconduction, different types of calcium phosphate were developed. Based on the Ca/P atomic ratios of 1.5 to 2, calcium phosphates such as hydroxyapatite, tricalcium phosphate and Whitlockite are widely used. Further, a variety of applications of these materials are in use to enhance bioactivity as coatings, to fill and heal bone defects as cements and to control the porosity and biocompatibility as scaffolds [9]. β-phase form of tricalcium phosphate (β-TCP; Ca_3_(PO_4_)_2_) has been extensively investigated and widely used clinically because of its similar chemical composition to the apatite naturally present in bone tissue [10]. Although β-TCP demonstrates various favorable characteristics for its clinical use, it is difficult to deliver to the target site and hard to compact adequately. To overcome the difficulty in shaping the material, a combination of different biomaterials has become a major area of research. Several polymers such as poly(l-lactic acid), PCL, poly(lactide-co-glycolide) and poly(3-hydroxybutyrate) have been reported for their potential to improve the handling properties of β-TCP [11,12,13,14].

PCL is a semicrystalline linear aliphatic polyester with a high degree of crystallinity and hydrophobicity, and it has been widely studied in tissue engineering due to its high level of biocompatibility and biodegradability [15]. It has already been approved for use, along with a range of medical and drug delivery devices. In this study, we evaluated the use of 25% PCL as an additive to improve the mechanical and osteoconductive properties of the β-TCP in terms of physicochemical analysis; static compression test, in vivo study and bone histomorphometric were performed. The formulated combination of ceramic scaffold PCL–TCP is considered a promising strategy for designing useful scaffolds for bone grafting in clinical practice.

## 2. Materials and Methods

### 2.1. Preparation of PCL–TCP Composites

The PCL–TCP composites were prepared from the combination of PCL (inherent viscosity: 1.0–1.3 dL/g, Sigma-Aldrich, St. Louis, MO, USA) and β-TCP (0.25–0.5 mm, Wiltrom Co. Ltd, Hsinchu County, Taiwan) with a biocomposite weight proportion of 1:3. PCL was stirred vigorously in dichloromethane (CH_2_Cl_2_, Sigma-Aldrich, St. Louis, MO, USA) for 2 h, followed by mixing with TCP for 2 min. Sodium chloride particles (Sigma-Aldrich, St. Louis, MO, USA) were then incorporated into the suspension. The ratio of TCP/PCL/sodium chloride was 3/1/17. Finally, the dispersion was cast into a Ti mold and air-dried for 24 h to allow the solvent to evaporate. Subsequently, the TCP/PCL/sodium chloride powders were immersed in deionized water for 6 h, and the water was changed approximately every 2 h at room temperature in order to leach the salt out. The PCL–TCP powder was dried for 24 h in an oven at 30 °C.

### 2.2. Physicochemical Analysis

The physicochemical properties of the scaffolds were evaluated based on the porosity, swells, flow rate and pore size analysis. SEM (SEM, JEOL 5565, Tokyo, Japan) was used to examine the microstructure of PCL–TCP powder. Pore size distribution and porosity were determined by analyzing the SEM images using the ImageJ software (NIH, Bethesda, MD, USA). Ten fields were randomly selected for each SEM image. In total, five replicates were conducted.

### 2.3. Setting Time and Static Compression Test

PCL–TCP was heated to 70 ℃ in a water bath. The setting time of PCL–TCP was then measured by Vicat Needle (HCH-122, Hsinchu County, Taiwan) on a Material Test Equipment (Instron® ElectroPuls™ E10000, Norwood, MA, USA) at 37 °C. The mean setting time was recorded from three individual batches.

Compression test was performed to examine the compressive strength (MPa) in response to an applied compression load using a universal testing system (Instron® ElectroPuls™ E10000, Norwood, MA, USA). A constant extension of 1.3 ± 0.3 mm/min was applied up to a strain value of 0.1 mm/min. The resulting force displacement response was recorded continuously.

### 2.4. Surgical Procedures and Implantation

**Figure 1:**
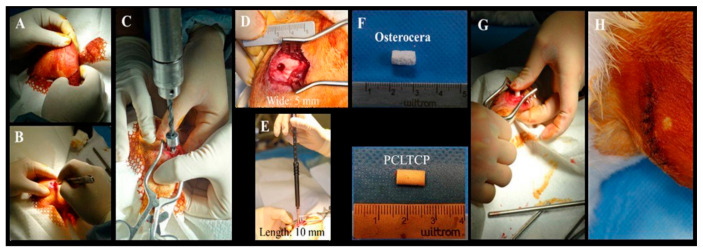
Surgical procedure for creating lateral femur lesion site and implantation. Both hind legs of each rabbit were shaved and a 2.5 cm skin incision was made (**A**,**B**). The skin was then retracted laterally to allow lateral arthrotomy on the stifle joints (**C**). A surgical drill bit and a stopper set were used to create a bony defect of 5 mm in diameter and 10 mm in depth in the femur at the midpoint of the lateral condyle from the lateral fabella to the anterior portion of lateral trochlea (**D**,**E**). The target sites were then filled with appropriate bone substitute cylindrical filaments, followed (**F**,**G**) by endodermis and muscle tissue being stitched using absorbable suture and skin stitches with nylon suture (**H**).

Eighteen New Zealand rabbits (BioLASCO Taiwan Co., Ltd., Taipei, Taiwan) with a weight of at least 2.8 kg were used in this study. The investigation was approved by Institutional Animal Care and Use Committee (IACUC) of MASTER LABORATORY Co., Ltd. (IACUC approval number: MS20161101). All experimental procedures were conducted in accordance with National Institutes of Health Guidelines. The rabbits were divided into three groups according to the implantation time (Table 1). The rabbits were anesthetized by intramuscular injection of a mixture of 0.5 mL/kg Zoletil (Virbac, France) and 0.5 mL/kg Rompun (BAYER KOREA LTD., Seoul, KOREA) and 2% Xylocaine (Recipharm Monts, Monts, France) subcutaneously. Both hind legs of each rabbit were shaved and a 2.5 cm skin incision was made (Figure 1A,B). The skin was then retracted laterally to allow lateral arthrotomy on the stifle joints (Figure 1C). A surgical drill bit and a stopper set were used to create a bony defect of 5 mm in diameter and 10 mm in depth in the femur at the midpoint of the lateral condyle from the lateral fabella to the anterior portion of lateral trochlea (Figure 1D,E). The target sites were then filled with appropriate bone substitute cylindrical filaments, followed (Figure 1F,G) by endodermis and muscle tissue being stitched using absorbable suture and skin stitches with nylon suture (Figure 1H). The PCL-TCP bone substitutes were prepared by a heating device and injected into the irregular bone damaged during the surgery to facilitate the process. All rabbits were monitored before the designated implantation observation time at 4, 12 and 24 weeks when the left and right femora were collected. Tissues were placed in 10% formalin until further analyses.

### 2.5. Radiological and Histomorphometric Analyses

The X-ray of the femoral condyles obtained at the designated implantation time were taken using the Siemens Arcadis Varic C-arm system (SOMA TECH INTL, Bloomfield, CT, USA). The femurs for undecalcified sectioning were dehydrated and embedded in polymethyl methacrylate (PMMA) (Merck, Germany). A section cut was made in the sagittal plane of the target region and surrounding bone with 5–10 mm thickness and 5 mm from the top of the defect at the lateral condyle for each specimen. Sections with a final thickness of 5 μm were obtained and stained with Masson–Goldner trichrome for histomorphometric analyses. The sections were examined under a light microscope (Olympus BX43) to identify the newly formed bone and the implant. Area measurements were made using the “Tile Overlapping Image” function in Media Cybernetics’ Image Pro Plus (IPP) program (Image Pro Plus, Media Cybernetics, Inc., Rockville, MD, USA).

### 2.6. Statistical Analysis

Data are presented as mean ± standard deviation. Statistical differences were determined by one-way analysis of variance with Tukey’s HSD post hoc tests to compare the mean changes between difference composite scaffolds. *p*-values of <0.05 were considered statistically significant.

## 3. Results

### 3.1. Morphology and Mechanical Analysis

**Figure 2:**
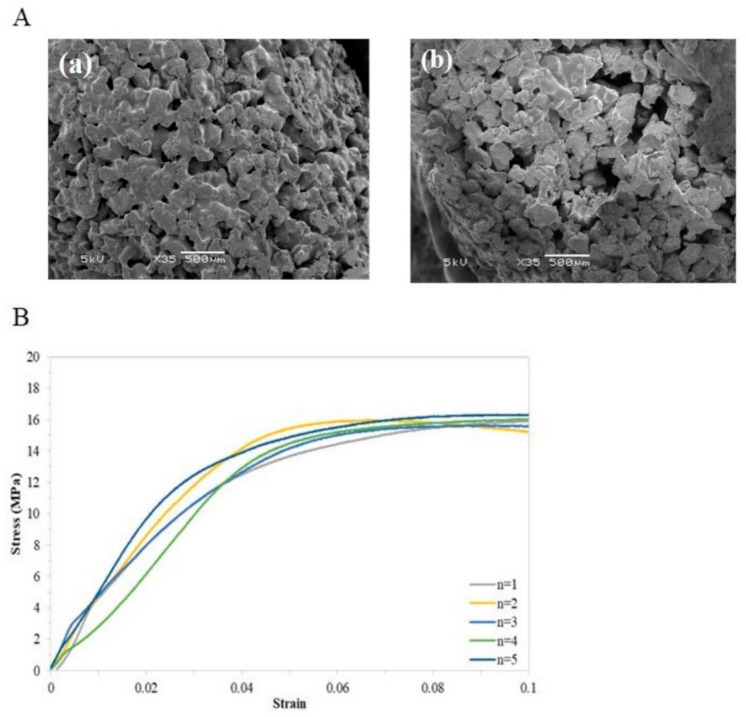
Physicochemical characterization of PCLTCP composite. (**A**) SEM images of: (**a**) top and (**b**) cross section view of PCL-TCP scaffolds. (**B**) Stress–strain curve obtained by static compression test.

The physicochemical characterization of the β-TCP (Osteocera^TM^) and PCL–TCP focused on the pore size, porosity, compressive strength and the material setting time. Results showed that the designed scaffolds presented a well-defined architecture with uniform pore distribution. The pore size of the PCL–TCP scaffolds was 235.28 ± 113.50 µm, which was smaller than the β-TCP (463 ± 88.75 µm, *p* < 0.001), while the porosity percentage was 43.00 ± 15.98% and 83.05 ± 1.52%, respectively (*p* < 0.001) (Table 2). A significant increase in the compression load of PCLTCP was observed at a strain of 0.1 (PCL–TCP: 15.10 ± 0.53 MPa vs β-TCP: 0.85 ± 0.19 MPa, *p* < 0.001). PCLTCP was further tested for a maximum setting time of 85 s, and the final curing time was recorded as 72.6 ± 8.5 s (Table 2 and Figure 2B). Figure 2A showed SEM images (top and cross section views) of PCL–TCP scaffolds with a uniform pore distribution.

### 3.2. Clinical Observation and Gross Bone Morphology

**Figure 3:**
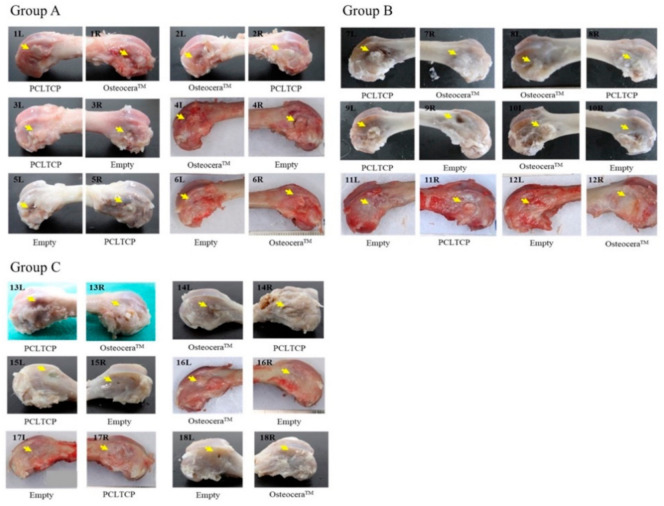
Gross bone morphology at 4-, 12- and 24-weeks post-implantation with PCL–TCP. The femurs with appropriate treatments of all 18 rabbits at designated implantation observation time points are shown. Yellow arrows indicate the implant sites.

All animals were carefully monitored throughout the study period with body weights taken daily. The average body weight increases were 249.5 ± 53.8 g, 490.2 ± 74.8 g and 608.3 ± 45.3 g in groups A, B and C, respectively. There were no differences in intragroup body weight, suggesting that different treatments did not affect the growth of the animals. All surgical wounds were closed and healed with no signs of infections at the time of the final evaluation. There were no complications observed throughout the study period. The appearances of the femurs obtained from all eighteen rabbits are shown in Figure 3. All implant sites were successfully healed without any abnormalities.

### 3.3. Radiographic Analyses

**Figure 4:**
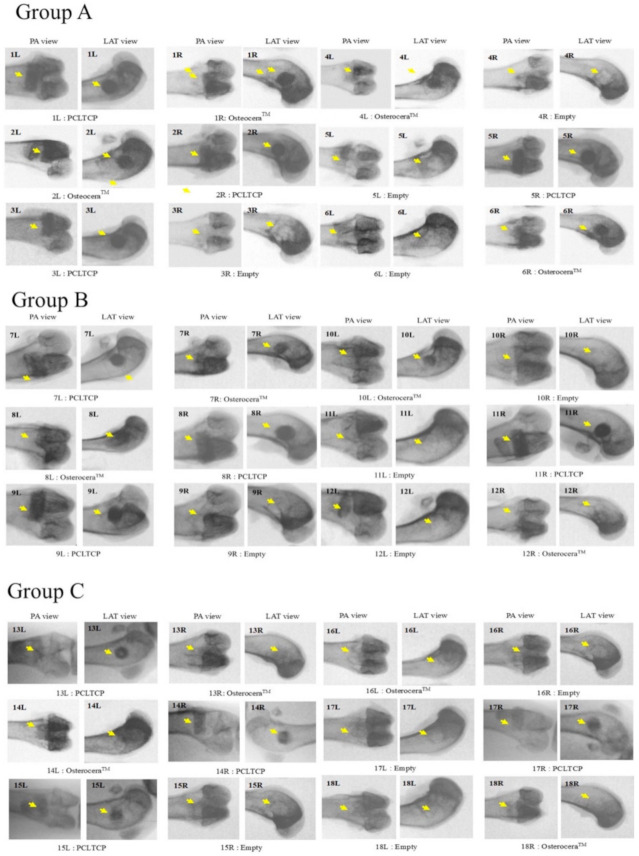
Radiographic images at 4- 12-, and 24-weeks post-implantation with PCLTCP. X-ray was taken from all the femurs with appropriate treatment of eighteen rabbits at designated implantation observation time points.

X-ray images were taken to examine bone regeneration at different implantation time points (Figure 4). Cylindrical filaments of PCLTCP and β-TCP were clearly seen at the implant sites at 4 weeks post-implantation, while part of the boundaries between implant and surrounding bones were indistinct in β-TCP (2/4 specimen) but not in PCLTCP filled implant sites at 12 weeks. We observed smeared and less distinguishable boundaries in all the implant sites from PCL–TCP and β-TCP at 24 weeks post-implantation, indicating bone graft degradation of PCL–TCP and β-TCP after 24 weeks. The injury in the control group remained throughout the study period.

### 3.4. Histomorphometric Examinations

**Figure 5:**
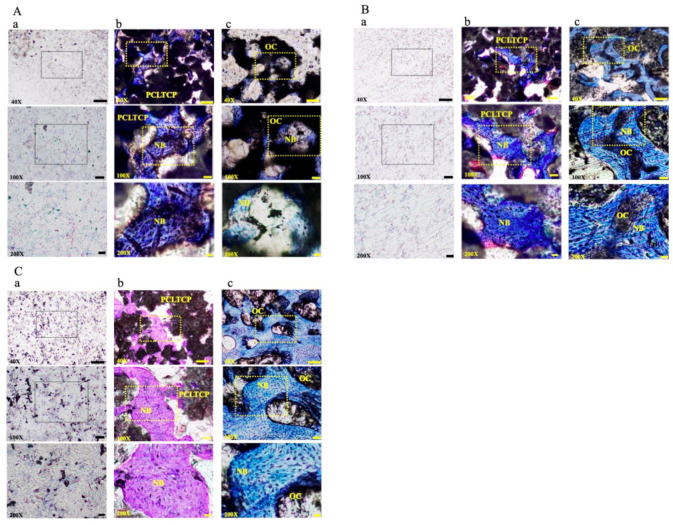
Representative photomicrographs from PCL–TCP implants at (**A**) 4, (**B**) 12 and (**C**) 24 weeks post-implantation. The treatments in each implantation observation time group are labelled as (**a**) empty, (**b**) PCL–TCP and (**c**) β-TCP (OC). NB represents newly formed bone. (Scale bar = 200 μm in 40×, 50 μm in 100×, 20 μm in 200×).

The clinical effectiveness of PCLTCP and β-TCP were harvested at each implant site for histomorphometric evaluations (Figure 5 and Table 3). The results showed that PCLTCP possessed the same biological affinity as β-TCP without adverse effects one month after rabbit femur condyle implantation. The newly formed bone area (NFBA) of the PCLTCP was significantly increased at 24 weeks compared to 4 weeks (*p* < 0.001). However, no statistical significance in percentage of NFBA between the PCLTCP and β-TCP was observed at 4 (*p* = 0.219), 12 (*p* = 0.139) and 24 weeks (*p* = 0.331). The boundary of the implants showed no surrounding soft tissue. The growth of newly formed bone was observed inside the materials. After three to six months, bone cavities were filled with new bone tissue and then reconstructed into secondary bone structure with a cancellous-like bone structure. Data suggested that the composite materials were resorbed and replaced by newly formed bone tissue. Both PCL-TCP and β-TCP demonstrated equivalent effects on biological affinity and osteoconduction property at 4-, 12- and 24-weeks post-implantation.

## 4. Discussion

The aim of our study was to fabricate an injectable and mechanically strong bone substitute that can be better applied to irregular bone defects during surgery. We have successfully demonstrated the injectability of PCL–TCP composite material, and characterized the mechanical properties of a PCL–TCP composite and its osteoconduction property following the implantation in rabbit bone injury model. The fabricated PCL–TCP provides higher compressive strength and better shaping characteristics than β-TCP, and enhances new bone growth from residual bone into the cancellous bone-like structure. The newly formed bone that connects both ends of the injury could compensate for the loss of mechanical strength due to degradation of β-TCP scaffolds [16]. The void left by β-TCP dissolution additionally increases the available surface area. Physiologically, β-TCP degrades quickly through both osteoclastic resorption and material dissolution [10,17], hence promoting the bone regeneration process [18]. Although spontaneous bone regeneration has been reported in rabbit bone defect model [19], a superior new bone formation was observed in the groups treated with filling composites compared to the control group.

The mechanical property and setting time of the hardened materials are also important indexes for bone repair in clinical practice, as setting time gives an advantage for the surgical operation with more time. Huang et al. incorporated different concentrations of β-TCP into PCL, with the aim of developing injectable and highly flexural strength materials [20]. They have shown that PCL containing a higher ratio of β-TCP resulted in declining mechanical strength but longer setting time. Ramay et al. combined β-TCP and hydroxyl apatite (HA) to fabricate a biodegradable nanocomposite porous scaffold that contained a compressive strength of 9.8 ± 0.3 MPa and stiffness of 1.72 ± 0.02 kN/m [21]. Recently, a composite of poly (propylene fumarate) (PPF) and β-TCP as a potential bone repair material with a maximum compressive strength of 133 ± 6 MPa and curing temperature of 54.7 ± 1.69 °C was also developed [22]. In yet another study, a composite of hydroxyapatite and β-TCP was mixed with polycaprolactone (PCL) to improve mechanical properties [23]. PCL-based β-TCP has been reported to have high mechanical strength and low degradation rate, which is considered a promising strategy for long-term hard tissue engineering [24].

The quality of bone integration is related to pore size and porosity as a function of structural permeability and biomechanics [25]. β-TCP is more hydrophilic than PCL by acting as a conductor to facilitate water diffusion [26]. The pore size and porosity percentage determine the solvent convective flow and describe the capacity of composites for bone substitution in terms of solutes and nutrient transportation [27]. The reported minimum pore size for a bone substitute is 100 μm [28]; however, the recommended pore size for great osteogenesis is over 300 μm [29,30]. Although PCL–TCP used in our study had a relatively small pore size and porosity, our data demonstrated equivalent effectiveness on osteoconduction property compared to the commercially available β-TCP scaffold (Osteocera^TM^). Results implied the potential of 25% PCL with heterogeneous pore size β-TCP scaffolds for rapid bone ingrowth.

The primary limitation in this study was the sample size of animals and the assessment of the PCLTCP degradation. The remaining implants of PCLTCP were 36% while β-TCP showed 23% left after 24 weeks implantation in rabbit femur. Previous in vivo study demonstrated 33% degradation of PCL-based β-TCP scaffold from 6 to 9 months after implantation in canine mandible [31]. More investigation is necessary for long-term observation of degradation of scaffolds and associated inflammatory response. Even with these limitations, the findings showed favorable and acceptable potential for further clinical implementation on patients using PCL–TCP scaffolds.

## 5. Conclusions

This study aimed to develop an injectable and mechanically strong bone substitute, and investigate the efficacy of PCL–TCP scaffolds for bone regeneration in rabbit defect model. Results were compared against β-TCP (Osteocera^TM^), a commercially available composite, which is commonly used in the clinical setting. PCL–TCP was proven to be as effective as the commercially available β-TCP scaffold in terms of the percentage of NFBA measured by histomorphometric analysis. The incorporation of 25% PCL with β-TCP leads to a relatively smaller pore size and porosity in scaffolds and higher compression load with good shaping characteristics, which also contributes to the clinical effectiveness of the in vivo model. Future work should further explore the bio-affinity, adaptability and the rate of desired degradation of PCL–TCP composite.

## Figures and Tables

**Table 1:** Grouping, treatment details and implantation time of each rabbit.

Group	No.	Treatment		Implantation Time (Week)
		Left	Right	
A	1	PCLTCP	β-TCP	4
	2	β-TCP	PCLTCP	4
	3	PCLTCP	Empty	4
	4	β-TCP	Empty	4
	5	Empty	PCLTCP	4
	6	Empty	β-TCP	4
B	7	PCLTCP	β-TCP	12
	8	β-TCP	PCLTCP	12
	9	PCLTCP	Empty	12
	10	β-TCP	Empty	12
	11	Empty	PCLTCP	12
	12	Empty	β-TCP	12
C	13	PCLTCP	β-TCP	24
	14	β-TCP	PCLTCP	24
	15	PCLTCP	Empty	24
	16	β-TCP	Empty	24
	17	Empty	PCLTCP	24
	18	Empty	β-TCP	24

**Table 2:** The physicochemical characterization of the composite scaffolds.

	PCL-TCP	β-TCP
Product name	“Wiltrom” Bitrans Bone Graft Substitute	“Wiltrom” Osteocera Bone Graft Substitute
Composite	β-tricalcium phosphate, β-TCP 75% polycaprolactone, PCL 25%	β-tricalcium phosphate, β-TCP > 95%
Pore size From SEM	235.28 ± 113.50 (μm)	463 ± 88.75 (μm)
Porosity From SEM	43.00% ± 15.98%	83.05% ± 1.53%
Compressive strength	15.104 ± 0.530 (MPa)	0.85 ± 0.19 (MPa)
Setting time	72.6 ± 8.5 (s)	NA

**Table 3:** Summary of histomorphometric analysis. Values are presented as mean ± standard deviation.

Group/Treatment		Remaining Implant (%)	Void Space Area (%)	Newly Formed Bone Area (%)
A	PCLTCP	50.22 ± 7.61	31.06 ± 7.36	18.72 ± 4.48
	β-TCP	51.73 ± 8.22	26.57 ± 6.92	21.70 ± 7.44
	Empty	NA	98.00 ± 0.92	2.00 ± 0.92
B	PCLTCP	53.72 ± 4.61	18.25 ± 5.66	28.03 ± 4.82 *
	β-TCP	32.94 ± 6.96 *	35.41 ± 10.72	31.32 ± 5.91 *
	Empty	NA	98.05 ± 0.92	1.95 ± 0.92
C	PCLTCP	36.51 ± 6.84 *	28.80 ± 4.04	34.69 ± 6.06 *
	β-TCP	23.02 ± 7.22 *	39.87 ± 11.14	37.11 ± 10.65 *
	Empty	NA	98.10 ± 0.76	1.90 ± 0.76

Asterisks (*) indicate significance (*p* < 0.05) compared with Group A.

## Data Availability

The data presented in this study are available on request from the corresponding author.
